# Salivary immunoglobulin levels and periodontal indices in Brazilian children with and without type 1 diabetes

**DOI:** 10.1590/1807-3107bor-2024.vol38.0043

**Published:** 2024-05-13

**Authors:** Thyciana Rodrigues RIBEIRO, Sara Maria SILVA, Renata Asfor Rocha Carvalho MARTINS, Cláudia Ferreira SANTOS, Paulo Goberlânio de Barros SILVA, Adriana Costa e FORTI, Fábio Wildson Gurgel COSTA, Manassés Claudino FONTELES, Cristiane Sá Roriz FONTELES

**Affiliations:** (a) Universidade Federal do Ceará – UFC, School of Dentistry, Department of Clinical Dentistry, Fortaleza, CE, Brazil.; (b) Universidade Estadual do Ceará – UFC, Higher Institute of Biomedical Sciences, Fortaleza, CE, Brazil.; (c) Centro Universitário Christus – Unichristus, Division of Oral Pathology, Fortaleza, CE, Brazil.; (d) Universidade Federal do Ceará – UFC, Department of Clinical Medicine, Fortaleza, CE, Brazil.

**Keywords:** Immunoglobulins, Dental Plaque Index, Periodontal Index, Diabetes Mellitus, Type 1

## Abstract

This cross-sectional study evaluated the association between salivary immunoglobulins, plaque index, and gingival index in Brazilian children with and without type 1 diabetes mellitus (DM1). The Strengthening the Reporting of Observational Studies in Epidemiology (STROBE) checklist for the reporting of observational studies was followed. The DM1 group had 38 children, and an equal number of volunteers matched by sex and age were recruited as controls. Clinical examination was performed for plaque index and gingival index determination. Non-stimulated whole saliva was collected. Concentrations of IgA, IgG, and IgM were determined by ELISA test. Data were tested by the Kolmogorov-Smirnov, Mann-Whitney, and Spearman tests and a multiple linear regression model (p<0.05) was performed. Gingival index was higher in the Control (DM1: 0.16±0.17; Control: 0.24±0.23, p=0.040). In DM1, there was a correlation between IgA and age (rho=0.371, p=0.024), IgM and IgG (rho=0.459, p=0.007), and IgM and gingival index (rho=0.394, p=0.014). In DM1, multiple linear regression showed that age (p=0.041; β=0.363), gingival index (p=0.041; β=0.398), and plaque index (p=0.008; β=-0.506) were good predictors of IgA levels in saliva. Thus, IgA was the only researched immunoglobulin that was directly associated with plaque and gingival indices in Brazilian children with DM1, but not in control subjects.

## Introduction

Type 1 diabetes mellitus (DM1) is a growing public health problem that accounts for 5–10% of all diabetes cases worldwide.^1^ Brazil has the third highest prevalence of DM1 in children and adolescents (92.3 million), after India (229.4 million) and the United States of America (157.9 million), and an annual incidence of 8.9 million in this age group.^2^

Oral complications are common in DM1, especially in those with decompensated metabolic disease.^3^ Diabetes is the most important systemic disease that adversely affects periodontal tissues,^4^ with gingivitis and periodontitis considered well-established sequelae of this condition.^5^ Parameters such as the gingival index, which assesses the marginal and interproximal gingival condition,^6^ and the plaque index, which measures the presence of soft debris and deposits on tooth surfaces,^7^ are frequently used to assess periodontal health in these patients.^8,9^

Elevated immunoglobulin levels have also been strongly associated with the development and progression of inflammatory diseases.^10^ IgA deficiency in the blood has been observed in individuals with DM1 and is usually accompanied by normal or increased serum levels of IgG and IgM.^11^ Furthermore, Ardawi et al.^12^ found a positive correlation between serum levels of IgA and IgG and glycated hemoglobin (A1C) in patients with DM1, suggesting that metabolic control can influence the humoral response and the synthesis of immunoglobulins. The quantification of immunoglobulin levels in diabetics can therefore improve our understanding of immune dysfunction.^13^

Determination of salivary components in diabetes is also important for describing and understanding the oral findings in this condition, ^14,15^since systemic diseases such as DM1 can also compromise the function of the salivary glands and influence the quantity and quality of saliva produced.^16,17^ Compared to healthy controls, salivary concentration of IgA, IgM, and IgG did not appear to be altered in individuals with DM1.^18^ However, it is worth noting that, of the studies included in this systematic review with meta-analysis, only two, Belazi et al.^19^ and Javed et al.,^20^ evaluated patients younger than 20 years of age (4–17 and 10–19, respectively).

The relationship between levels of IgA, IgG, and IgM in saliva and plaque and gingival indices has already been investigated in systemically healthy children (3 to 12 years old) with and without gingivitis.^21^ Analysis of the relationship between immunoglobulin levels and clinical parameters of gingivitis showed that there was a direct correlation between IgM and gingival index (p < 0.05) in children with gingivitis. However, in children with DM1, this relationship has not yet been elucidated. To date, only the study by Wang et al.^22^ compared plaque and gingival indices and salivary factors (pH, buffering capacity, glucose, total protein, immunoglobulins, lysozyme, and lactate dehydrogenase) in children (7 to 15 years old) with and without DM1, but did not investigate possible associations between these variables. This gap in children with DM1 must thus be clarified.

The null hypothesis of this study was that there is no relationship between salivary immunoglobulins and periodontal indices in pediatric patients with DM1. Thus, the aim of the present study was to evaluate the association between salivary immunoglobulins (IgA, IgG e IgM), plaque index, and gingival index in children with and without DM1.

## Methodology

The Strengthening the Reporting of Observational Studies in Epidemiology (STROBE) checklist was followed in this study.

### Study design and ethical considerations

This was a comparative cross-sectional study. The present study was conducted ethically in accordance with the Declaration of Helsinki (World Medical Association) and approved by the Ethics Committee in Research Involving Human Beings of the Federal University of Ceará (protocol # 1.020.102), following the rules and regulations of the National Health Council. The parents or legal guardians of all participants signed an informed consent form before the child’s enrollment in the research.

### Sample calculation and patient recruitment

A sample calculation was performed using an independent samples t-test based on the gingival IgA levels of patients with periodontal disease and diabetes (11.43 ± 1.51 mg/dL) compared to patients without diabetes (10.16 ± 1.58 mg/dL )^23^ with 32 patients per group, a power of 90%, and a confidence interval of 95%. However, to account for losses, the sample was increased by 20% getting a final sample of 38 patients per group. Based on the IgA concentration in diabetic patients older than seven years observed in this study (59,200 ± 4,960 ng/mL) and controls (48,469 ± 4,891 ng/mL), a power of 100% was estimated for a sample of 38 patients per study group to reject the null hypothesis.

Thus, 38 children of both sexes, 2–10 years old and diagnosed with DM1 were selected to participate in the study. These children were spontaneously invited and examined in 2019 during routine consultations with the multidisciplinary team of the Integrated Center for Diabetes and Hypertension (Fortaleza, Brazil). Data collection was performed in 2019 and 2020. The eligibility criteria were: a) diagnosis of DM1; b) non-use of any medication that could alter salivary flow and/or composition; and c) absence of associated comorbidities at the time of study entry. The first 38 children who attended the service and met the eligibility criteria and whose parents consented to the participation in the study were selected. Such children made up the DM1 group. For the control group, the first 38 children without DM1 who attended the service at the pediatric dentistry clinic of the Dentistry course of the Faculty of Pharmacy, Dentistry and Nursing of the Federal University of Ceará in Fortaleza were selected, matched by gender, age and socioeconomic profile to children with diabetes, without comorbidities, meeting the previously defined eligibility criteria, and whose parents consented to participate in the study. Data obtained from these healthy participants were used for comparison.

Currently, diabetes may be diagnosed based on plasma glucose criteria, either fasting plasma glucose (FPG) value or the 2-h plasma glucose (2-h PG) value during a 75-g oral glucose tolerance test (OGTT) or A1C criteria.^24^ The criteria for diagnosing diabetes are: FPG ≥ 126 mg/dL (7.0 mmol/L) or 2-h PG ≥ 200 mg/dL (11.1 mmol/L) during OGTT or A1C ≥ 6.5% (48 mmol/mol) or a random plasma glucose ≥ 200 mg/dL (11.1 mmol/L) in a patient with classic symptoms of hyperglycemia or hyperglycemic crisis.^24^

### Data collection

Initially, information concerning the general health status of the participants was obtained. Subsequently, gingival health was assessed using the plaque index, as described by Silness & Löe^7^ and the gingival index was assessed according to the study by Löe & Silness^6^. The clinical oral examination was carried out in the dental offices of the patients’ services, with adequate lighting (reflector), mouth mirror, and appropriate probe. The plaque index was recorded for each tooth in 4 areas (distobuccal, buccal, mesiobuccal, and lingual). The sum of the plaque indices of each tooth was divided by 4 to obtain the plaque index per tooth. The plaque index per individual was obtained by adding the plaque indexes per tooth, followed by its division by the number of teeth examined.^7^ The gingival index, in turn, was recorded for each tooth in 4 areas (distobuccal papilla, buccal margin, mesiobuccal papilla, and complete lingual margin). For each tooth, the gingival index scores of each area were added and then divided by 4, to obtain the gingival index per tooth. The score per individual was obtained by adding the scores per tooth and then dividing by the number of teeth.^6^ Teeth about to exfoliate, with pathological mobility, in eruption or with purulent exudate according to Romero et al.^21^ were not included. The gingival health was assessed by a single calibrated examiner with an intra-examiner Kappa of 0.81^26^ for both indices. The calibration was performed with 18 healthy children. The evaluators received training before the calibration. The interval between the evaluations was one week.

The collection of saliva for immunoglobulin evaluation was performed by a single researcher after gingival assessment in the dental offices of the services where the patients came from. A sample of unstimulated whole saliva was collected for each participant between 8 and 10 am to reduce possible circadian effects after a maximum of 2 hours of fasting (due to the risk of hypoglycemia in the group with DM1). Parents were asked to perform routine oral hygiene in the child one hour before collection. The volunteer remained at rest for 30 minutes and then saliva was collected with the aid of a plastic Pasteur pipette and stored in Eppendorf^®^ microtubes (Sigma – Aldrich Brasil LTDA, São Paulo, Brasil), followed by the addition of 5 μL enzyme inhibitor (Sigma – Aldrich Brasil LTDA, São Paulo, Brasil) to each 1 mL of collected saliva. The samples were kept and transported on ice to the laboratory for subsequent centrifugation at 12,000g for 10 minutes at 4°C, lyophilization of the supernatant, and storage at -80°C until analysis. This standardized protocol was used to control for environmental and circadian rhythm influences on the qualitative and quantitative salivary components.^26^

### Quantitative analysis of immunoglobulins – ELISA

Each saliva sample was divided into 3 parts to be analyzed individually, being subjected to the analysis of immunoglobulin titers using the enzyme-linked immunosorbent assay (ELISA), using saliva as the primary antibody and a human antibody component as the capture antibody. The following salivary assay protocols were used: human protein IgA, Abcam^®^, Cat. Num. ab137980; human IgG protein, Abcam^®^, Cat. Num. ab195215; and, human IgM protein, Abcam^®^, Cat. Num. ab137982 (Sigma – Aldrich Brasil LTDA, São Paulo, Brasil). Saliva and salivary standards were added to the wells followed by antibodies; wash buffer was used 5 times and stop solution was added to stop the reaction. Finally, the results were read in a spectrophotometer at 450 nm wavelength. All analyses were performed on a single day by the same researcher.

### Statistical analysis

Data analysis was performed using SPSS software (Statistical Package for the Social Sciences), version 20.0 for Windows^®^. Data were submitted to the Kolmogorov-Smirnov normality test, expressed as mean and standard deviation, and compared using the Mann-Whitney test (nonparametric data). Additionally, Spearman’s correlation was used to correlate clinical parameters and immunoglobulin profile. The variables that were correlated with the immunoglobulin profile were selected for the multiple linear regression model. The ROC (receiver operating characteristic) curve was also used to determine sensitivity, specificity, and accuracy. A cut-off point of 7 years old was established because 7 was the age median. The significance level was p < 0.05 for all tests.

## Results

The sample consisted of 76 children, 38 with DM1 and 38 without the disease. All of them were residents of Fortaleza (Brazil). There was no statistically significant difference for age according to sex (Control group: 7.20 ± 2.17 for female and 6.78 ± 2.26 for male, p = 0.657; DM1 group: 7.15 ± 2.01 for female and 6.67 ± 1.97 for male, p = 0.544) and age of the two groups (7.00 ± 2.19 for DM1 and 6.92 ± 1.98 for Control, p = 0.785).

**Figure 1:**
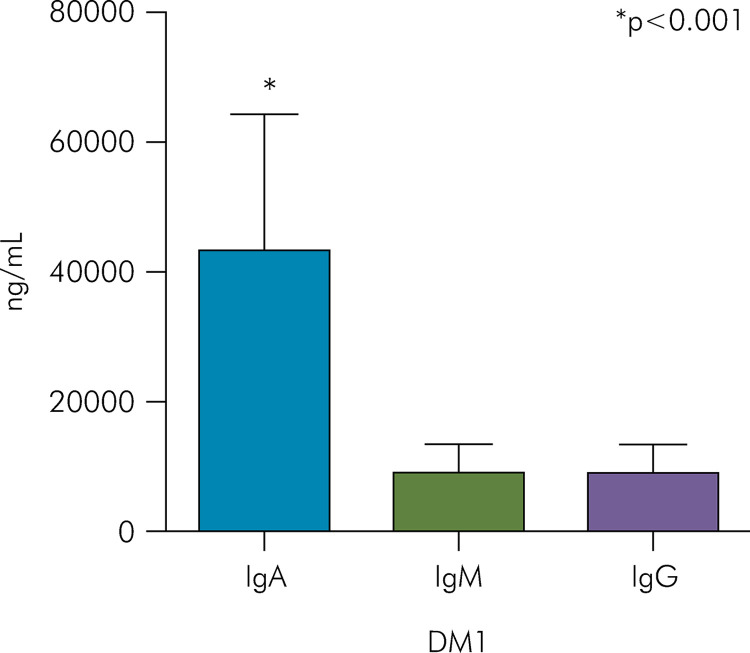
Comparison between immunoglobulin concentrations in the DM1 group (Mann-Whitney test; *p < 0.05).

Both the DM1 group and Control group had higher concentrations of IgA (43,005 ± 21,236 ng/mL and 43,857 ± 24,585 ng/mL) (Figure 1).

**Figure 2:**
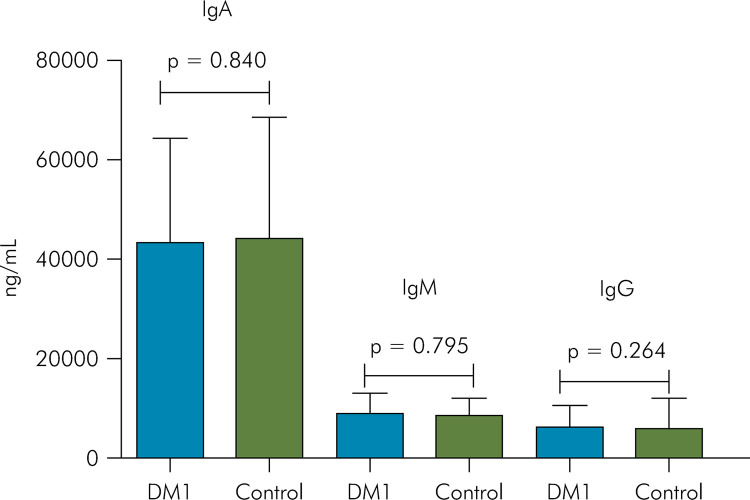
Comparison between DM1 and Control groups according to immunoglobulin concentrations (Mann-Whitney test; *p < 0.05).

There was no statistically significant difference between the groups for concentrations of IgA, IgM, and IgG (Figure 2).

There was no statistically significant difference between the groups for the gingival (0.17 ± 0.17 DM1 and 0.22 ± 0.20 Control; p = 0.145) and plaque indices (0.34 ± 0.32 DM1 and 0.22 ± 0.23 Control; p = 0.139) (Mann-Whitney test, p < 0.05). The mean gingival index in the Control group was 0.24 and in the DM1 group was 0.15. The median gingival index in the Control group was 0.19 and in the DM1 group was 0.10. The mean plaque index in the Control group was 0.22 and in the DM1 group was 0.34. The median plaque index in the Control group was 0.14 and in DM1 group was 0.23.

**Figure 3:**
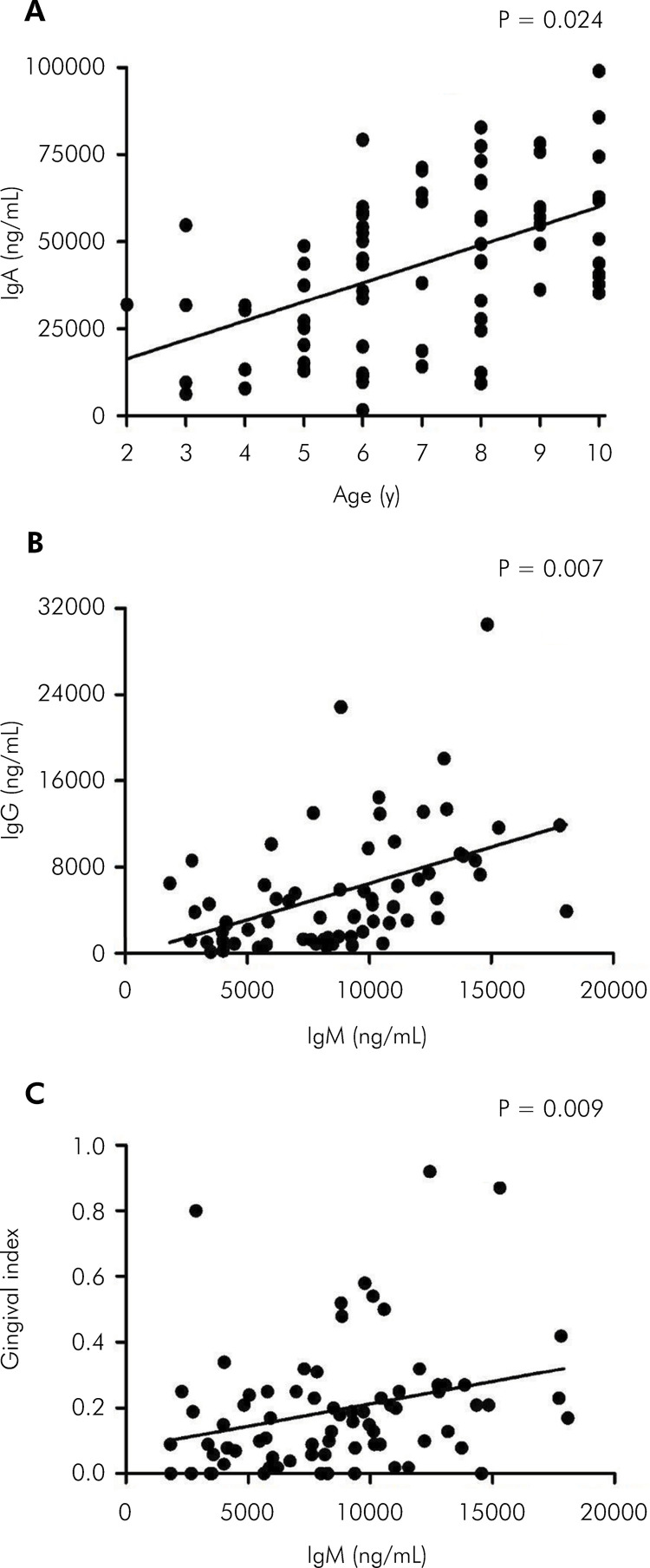
Correlation between IgA and age (A); IgG and IgM (B); and gingival index and IgM (C) in the DM1 group (Spearman’s test; *p < 0.05). (●) Control group; (▲) DM1 group.

In the DM1 group, there was a correlation between IgA and age, between IgG and IgM, and between gingival index and IgM (Figure 3).

Multiple linear regression showed positive correlations between age and IgA and age and IgM in the Control group, and between age and IgA and gingival index and IgA in the DM1 group. However, a negative correlation between plaque index and IgA in the DM1 group was found (Table 1).

**Table 1:** Salivary immunoglobulins, plaque and gingival indices and age in both groups.

Variable	Control		DM1	

	p-value	β ajustado	p-value	β ajustado
IgA (outcome)				
Plaque index	0.704	0.058	0.008*	-0.506
Gingival index	0.052	-0.307	0.041*	0.398
Idade	< 0.001*	0.677	0.041*	0.363
IgM (outcome)				
Plaque index	0.122	0.254	0.132	0.289
Gingival index	0.111	-0.258	0.268	0.223
Idade	< 0.001*	0.570	0.759	-0.056
IgG (outcome)				
Plaque index	0.565	0.120	0.468	-0.155
Gingival index	0.991	-0.002	0.876	0.036
Idade	0.153	0.264	0.091	0.367

Multiple linear regression; *p < 0.05.

**Figure 4:**
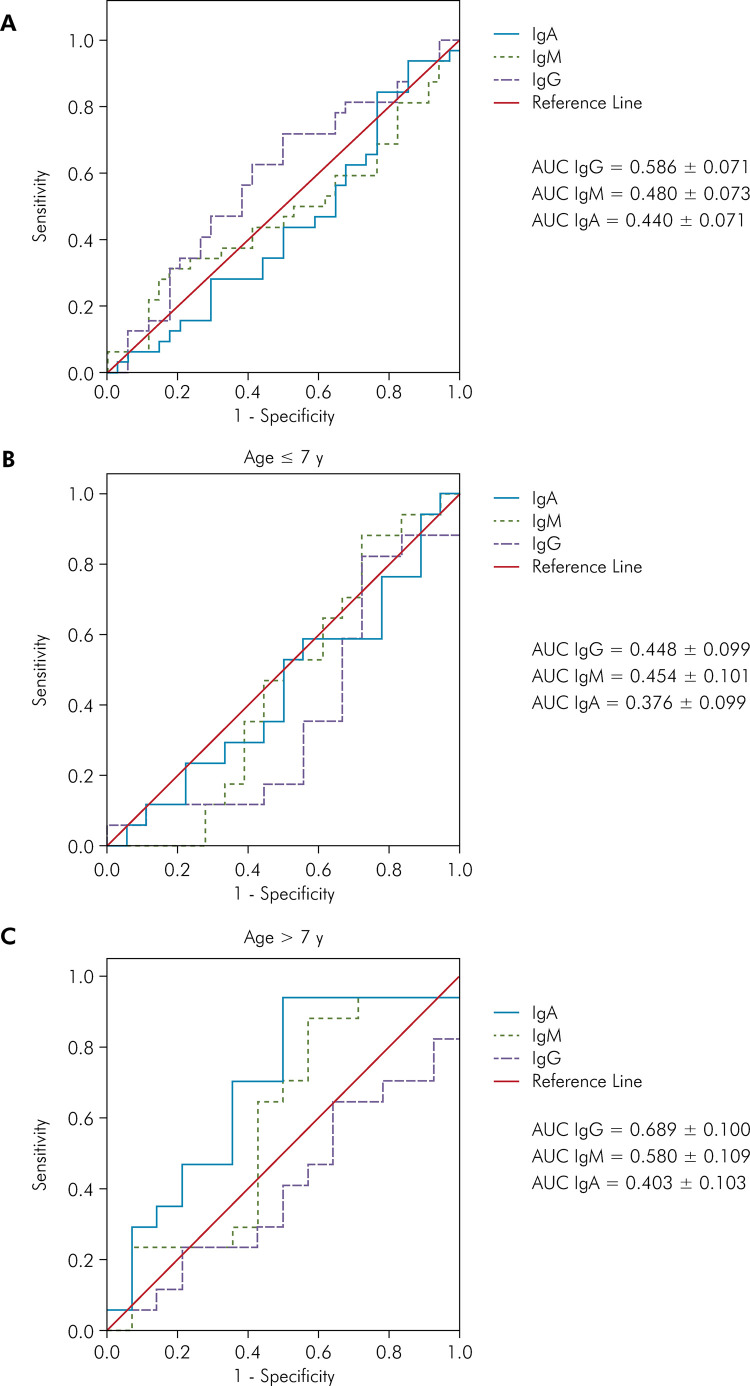
ROC curve for diagnosis of diabetes based on alterations in IgA, IgM and IgG levels (A) in children aged up to 7 years (B) and over 7 years (C) of age. A cut-off point of 7 years old was established because 7 was the age median.

The ROC curve for diagnosing diabetes based on changes in IgA, IgM, and IgG levels showed a large area under the curve for IgG, but it was not sufficient to establish a diagnosis. However, with a cut-off point of 7 years old, a greater area under the curve was observed for children over 7 years old than in children up to 7 years old (Figure 4).

Finally, children over 7 years old with an IgA concentration of up to 43,000 ng/mL had 88.2% specificity, and children over 7 years old with an IgG concentration of up to 3,200 ng/mL had 78.6% sensitivity (Table 2).

**Table 2:** Sensitivity and specificity of Immunoglobulins in predicting DM1 in children over 7 years old.

Over 7 years (DM1)	Sensitivity	Specificity	PPV	NPV	Accuracy
IgA (ng/mL)					
≤ 43,000*	47.1%	**88.2%**	80%	62.5%	67.6%
> 43,000					
IgM (ng/mL)					
≤ 9,000*	47.1%	70.6%	61.5%	57.1%	58.8%
> 9,000					
IgG (ng/mL)					
≤ 3,200	**78.6%**	35.3%	50%	66.7%	54.8%
> 3,200*					

*Cut-off point for diagnosis of DM1 in this sample. PPV, positive predictive value; NPV, negative predictive value.

## Discussion

In the present study, association and correlation analyses were performed showing important results for the DM1 group, including high levels of IgA in saliva, higher plaque index and lower gingival index compared to the Control group, and significant correlations between IgA and age, IgM and IgG, and IgM and gingival index.

The salivary IgA concentration of children with DM1 in the present study was higher than that of children without the disease, although this difference was not significant between the groups. Siudikiene et al.^27^ also observed high levels of this immunoglobulin when evaluating saliva of 63 pairs of children with and without DM1. Increased levels of salivary IgA in children with DM1 were also observed earlier by Belazi et al.^19^. Salivary IgA (s-IgA) is produced by plasma cells and inhibits the adhesion of microorganisms to mucosal surfaces. Increased levels of s-IgA in the oral cavity may indicate periodontal disease or oral candidiasis.^19^ Furthermore, according to Belazi et al.^19^, high levels of s-IgA combined with good oral health and a mild degree of dry mouth may indicate a prediabetic condition.

Although diabetes is recognized as a risk factor for periodontal diseases,^28-30^ in the present study, plaque and gingival indices did not differ significantly between groups. According to the systematic review with meta-analysis by Jensen et al.^28^, children and adolescents with DM1 are more likely to have elevated risk markers for periodontal disease (plaque [standardized mean difference - SDM 0.45; 95%CI [0.21–0.70]; p < 0.001] and gingival [SDM 0.51 95%CI [0.28–0.74]; p < 0.001] indices, bleeding on probing [SDM 0.61 95%CI [0.40–0.82]; p < 0.001], pocket depth [SDM 0.55 95%CI [0.22–0.87]; p < 0.001], and clinical attachment loss [SMD 0.54; 95%CI [0.29–0.78]; p < .001]) compared to their healthy peers. Zainal Abidin et al.^30^ also found a worse periodontal status in children and adolescents with DM1, SMD pooled [95%CI] for plaque index of 0.54 [0.20–0.87], gingival index of 0.63 [0.39,0, 87], clinical attachment loss of 0.79 [0.52–.05], and periodontal probing depth of 0.67 [0.23–1.11]. This has also been attributed to the elevated and prolonged inflammation of the periodontal tissue due to impaired immune function, leading to tissue destruction.^30^ However, it is worth emphasizing that when comparing periodontal studies, differences may be due to the design of the registry, type/number of sites evaluated, and periodontal probe used.^31^ Other factors such as diabetes duration and blood glucose may influence clinical outcomes.^9^

Rappone et al.^29^ also reinforced that studies on the potential effects of periodontal inflammation in children and adolescents with DM1 are incomplete. Despite the recent exponential increase in the number of studies on the association between periodontitis and diabetes, no general consensus has yet emerged on a causal effect of periodontal inflammation in T1DM. The authors concluded that their meta-analysis did not provide strong evidence that periodontitis is a significant risk factor for DM1, and the link between periodontal disease and DM1 does not appear to be as solid as the link with DM2.

Pachoński et al.^32^ observed similar resutls when comparing periodontal status (through plaque, proximal plaque, gingival, and modified sulcus bleeding indices) of healthy children, of children with compensated DM1, and of children with decompensated DM1, further suggesting the absence of an effect of metabolic control of diabetes on index values. However, a limitation of the study by Pachoński et al.^32^ was the small sample of patients, which resulted in a small margin of error. In addition, the threshold value of A1C was set at 7.5%. Ismail et al.^33^ also found a similar result for gingival index but observed that diabetic children had significantly higher plaque deposits and significantly higher mean plaque index when compared to healthy children.

In the DM1 group, an association between IgA and age was found. According to Romero et al.^21^, in the general pediatric population, the changes due to the onset of the mixed dentition phase from the age of 6 years lead to changes in the gingival crevicular environment, facilitating the colonization of microorganisms associated with periodontal diseases, thus increasing the antigenic challenge, which is reflected in higher levels of this immunoglobulin. Furthermore, it is important to consider that hormonal changes that begin before puberty can also affect the subgingival microbiota with increasing age.^34^

IgM and IgG antibodies work together in immediate and long-term protection against infections.^35^ Although the present study has demonstrated a direct relationship between such immunoglobulins in the DM1 group, there are no other studies on this association in children to date. In adults, Tenovuo et al.^16^ observed significantly elevated levels of IgG (p < 0.05) but not of IgM (p > 0.05). IgG and IgM are produced locally, for example, in the inflamed gingiva^36^ or are derived from serum via gingival clefts and/or salivary glands.^37,38^ In whole saliva, increased levels of immunoglobulins were detected in patients with periodontal disease.^39^

The significance of the positive correlation between IgM and gingival index in diabetics in the present study is still unclear. Romero et al.^21^, who evaluated levels of IgA, IgG, and IgM in saliva of healthy children aged 3 to 12 years with gingivitis, found a direct relationship between IgM and gingival index, and suggested two possible interpretations for this finding: the activation of IgM production as a response to an antigenic challenge leading to tissue inflammation or the increased exudate as a result of inflammation generates more IgM in saliva. Such hypotheses could eventually also apply to diabetic children.

Given the above, in the DM1 group, multiple linear regression showed that age, plaque, and gingival indices were good predictors of s-IgA levels. However, these variables were not good predictors of IgM or IgG levels in this population.

The present study included assessment of important risk markers for periodontal diseases that were validated as predictive for the amount of biofilm and the degree of gingival inflammation in a child.^40^ Furthermore, all measurements were performed by a single experienced examiner and complete data on periodontal markers and immunoglobulin analysis were obtained from all study participants. However, the present study also has some limitations. The cross-sectional design prevented the determination of causality between the variables evaluated. Also, the small sample size increases the margin of error.

## Conclusions

IgM showed a direct correlation with gingival index and IgA showed a significant association with plaque and gingival indices only in diabetic children.
